# Enhanced alleviation of aGVHD by TGF‐β1‐modified mesenchymal stem cells in mice through shifting MΦ into M2 phenotype and promoting the differentiation of Treg cells

**DOI:** 10.1111/jcmm.14862

**Published:** 2019-11-28

**Authors:** Ran Wu, Chuanxu Liu, Xiaohui Deng, Linjun Chen, Siguo Hao, Liyuan Ma

**Affiliations:** ^1^ Department of Hematology Xinhua Hospital Affiliated to Shanghai Jiao Tong University School of Medicine Shanghai China

**Keywords:** allogeneic haematopoietic stem cell transplantation, graft‐versus‐host disease, mesenchymal stem cell, transforming growth factor‐β1

## Abstract

Allogeneic haematopoietic stem cell transplantation (allo‐HSCT) is the only curative method in treating haematologic malignant diseases. Graft‐versus‐host disease (GVHD) is a common complication post–allo‐HSCT, which can be life‐threatening. Mesenchymal stem cells (MSCs) as an adult stem cell with immunoregulatory function have demonstrated efficacy in steroid resistant acute GVHD (aGVHD). However, the outcome of aGVHD treated with MSCs in clinical trials varied and its underlying mechanism is still unclear. TGF‐β1 is a potent cytokine, which plays a key role in immunoregulation. In the present study, we firstly transduced the lentivirus vector containing TGF‐β1 gene with mouse bone marrow‐derived MSCs. Then, we investigated the immunosuppressive effect of TGF‐β1 gene‐modified MSCs on lymphocytes in vitro and its preventive and therapeutical effects on murine aGVHD model in vivo. Murine MSC was successfully isolated and identified. TGF‐β1 was efficiently transduced into mouse MSCs, and high level TGF‐β1 was detected. MSC‐TGF‐β1 shared the same morphology and immunotypic features of normal MSC. In vitro, MSC‐TGF‐β1 showed enhanced immunosuppressive function on lymphocyte proliferation. In vivo, MSC‐TGF‐β1 showed enhanced amelioration on the severity of aGVHD both in prophylactic and therapeutic murine models. Finally, the macrophages (MØs) derived from MSC‐TGF‐β1–treated mice showed a remarkably increasing of anti‐inflammatory M2‐like phenotype. Furthermore, the differentiation of CD4+ CD25+ Foxp3+ Treg cells was significantly increased in MSC‐TGF‐β1–treated group. Taken together, we proved that MSC‐TGF‐β1 showed enhanced alleviation of aGVHD severity in mice by skewing macrophages into a M2 like phenotype or increasing the proportion of Treg cells, which opens a new frontier in the treatment of aGVHD.

## 1 BACKGROUND

Allogeneic haematopoietic cell transplantation (allo‐HSCT) remains an effective option in treating malignant disease of the haematopoietic system. However, graft‐versus‐host disease (GVHD) frequently happens after allo‐HSCT such that fatal GVHD offsets the benefit of allo‐HSCT and hampers development of this treatment.^1^, ^2^ Classically, three stages are involved in the development of aGVHD: firstly, tissue damage from conditioning regimen mediates the activation of antigen‐presenting cells (APCs); secondly, donor T lymphocytes are then activated by recipient antigens presented by host APCs; thirdly, donor T lymphocytes attack targets tissues and cause damage.^3^ aGVHD that does not respond to first‐line corticosteroid therapy is associated with a high mortality rate of 90%.^4^ Mesenchymal stem cells (MSC) isolated from bone marrow were firstly described by Friedenstein^5^ as spindle‐shaped, fibroblast‐like cells with the potency of differentiating into bone and cartilage in vitro. Based on its capacity of self‐renewal and differentiation into tissues including bone, cartilage and adipose, MSC has been widely used in tissue engineering and repair.^6^, ^7^, ^8^ MSC can also regulate immunity both by secreting soluble factors and by influencing the biology of immune cells. It is particularly important that MSC expresses few HLA class I and no HLA class II molecules, allowing them to evade allogeneic immune response. This is the so‐called ‘immunoprivilege’, an interesting feature in MSC biology, which makes these cells extremely suitable for both autologous and allogeneic transplantation.^9^ Owing to these multiple characteristics, MSC has been extensively researched and clinically applied as second‐line therapy for aGVHD.^10^, ^11^ From the first study by Le Blanc et al^12^ who successfully adopted MSC in the treatment of aGVHD in 2004, the application of MSC in aGVHD has made considerable progress in pre‐clinical and clinical research.^13^, ^14^, ^15^ However, there are great discrepancies amongst different groups, which could be attributed to the highly variable features of MSC due to the different tissue derivations, culture/experimental conditions and the number of passages of MSC.^13^, ^16^, ^17^ As MSC alone is suboptimal for treatment of aGVHD,^18^ there is a compelling clinical need for novel approaches to enhance its therapeutic and immunosuppressive property. One rational approach is to combine cell and gene therapy to achieve a greater immunoregulatory effect, by genetically modifying MSC to enhance its activity against aGVHD.^19^

The TGF‐β family of cytokines is pleiotropic cytokines that play an important role in regulating immune responses.^20^ TGF‐β1 is the commonest and most studied amongst the three isoforms of TGF‐β (β1, β2, β3). As a well‐characterized immunosuppressive molecule, it can down‐regulate multiple immune responses and participate in the pathological process of immune disorders.^21^ TGF‐β1 can be secreted by MSC and plays a non‐redundant role in the immunomodulatory function of MSC.^22^, ^23^ Sławomira KyrczKrzemień showed that low level of TGF‐β1 probably being one of the factors contributing to the development of acute GVHD. On the other hand, chronic GVHD symptoms seem to correlate with high TGF‐β1 mRNA expression and its serum concentration in patients who underwent bone marrow transplantation for myeloid leukaemia.^24^

Taken together, these reports indicate that both MSC and TGF‐β1 are potentially active against aGVHD. In this report, we put forward the hypothesis to apply TGF‐β1 gene‐modified MSC in treating aGVHD for the first time. Firstly, we transduced TGF‐β1 into mouse bone marrow‐derived MSC (MSC‐TGF‐β1), checked expression and production level of TGF‐β1, and characterized the immunophenotypic profile of MSC‐TGF‐β1. Secondly, we investigated its inhibitory function on T lymphocyte proliferation in vitro, as T lymphocytes are the main mediators of aGVHD. Thirdly, we examined the in vivo prophylactic and therapeutic efficacy of MSC‐TGF‐β1 on aGVHD with a mouse model. Finally, we studied the possible underlying mechanism of MSC‐TGF‐β1 in murine aGVHD. Based upon our comprehensive work, we paved a new way to better understand the characteristics of TGF‐β1 gene‐modified MSC and their role in treating aGVHD.

## 2 METHODS

### 2.1 Mice

Female BALB/c (BALB/c, H‐2d) mice and male C57BL/6 (B6, H2b) mice aged between 6 and 8 weeks were purchased from Slac Laboratory Animal Co, Ltd. All mice were housed under specific pathogen‐free conditions in the animal laboratory centre of Xinhua Hospital.

### 2.2 Isolation and culture of mouse MSCs

C57BL/6 mice were killed, and the marrow from femurs and tibias was extracted by repeatedly flushing with DMEM complete medium. The washing fluid was filtered through a 40‐μm strainer and centrifuged; the cells in the pellet were plated into culture flasks in low‐glucose DMEM medium supplemented with 10% FBS, penicillin (100 U/mL), streptomycin (100 U/mL) and incubated at 37°C in a humidified atmosphere containing 5% CO_2_ for 24 hours. After removal of non‐adherent cells, the cells remaining were cultured in fresh medium for another 48 hours as the first passage of MSC. Finally, MSC was passaged weekly and used for research after the third passage and before the 10th passage.

### 2.3 Differentiation of MSCs to adipocytes, osteoblasts and chondrocytes

Mesenchymal stem cells were cultured in completed medium supplemented with certain ingredients for 14‐21 days. Osteogenic (glutamine, ascorbate and β‐glycerophosphate), chondrogenic (dexamethasone, ascorbate, ITS + Supplement, sodium pyruvate, proline) and adipogenic (glutamine, insulin, IBMX, rosiglitazone) ingredients were added to the culture medium according to the manufacturer's instructions (Cyagen). The differentiated MSCs were fixed with 4% paraformaldehyde and stained with Alizarin red, alcian blue and oil red O, respectively, at proper times.

### 2.4 TGF‐β1 gene cloning and lentivirus production

Murine TGF‐β1 gene was amplified by polymerase chain reaction (PCR) performed with the following primers: forward: 5′‐ATGCCGCCCTCGGGGCTGC‐3′ and reverse: 5′‐GCTGCACTTGCAGGAGCGC‐3′. The purified TGF‐β1 gene fragment was cloned into the pHBLV‐CMVIE‐ZsGreen‐Puro plasmid (Hanbio) using EcoRI, XhoI, XbaI and BamHI restriction sites. Lipofiter™ (Hanbio) was used to cotransfect the plasmid containing the TGF‐β1 gene into 293T cells with a packaging plasmid (pSPAX2) and envelope plasmid (pMD2G). Lastly, we collected the virus particles from the cell supernatant and termed them as LV‐TGF‐β1. In addition, a control vector expressing GFP and puro was constructed and termed as LV‐GFP‐puro.

### 2.5 MSC transduction

Mesenchymal stem cell was seeded into 6‐well plates at a density of 2 × 10^5^ per well and transduced with LV‐GFP‐puro or LV‐TGF‐β1 at a multiplicity of infection (MOI) of 10 (the viral particles were previously quantified as 2 × 10^8^ CFU/mL) and cultured in 2 mL of DMEM complete medium supplemented with 5 μg/mL polybrene. After 24 hours of incubation at 37°C in a humid atmosphere contain 5% CO_2_, the media was changed into fresh complete medium with 15 μg/mL puromycin. The transduction efficiency was assessed 2 days later through observing under inverted microscope and quantifying by flow cytometry.

### 2.6 Flow cytometry

The cells for flow cytometry were collected, washed and stained according to the manufactures' instructions. The antibodies employed in the flow cytometric analysis included anti‐CD11c‐PE, anti‐Sca‐1‐PE, anti‐CD45‐PE, anti‐PDGFR‐α‐PE, anti‐CD29‐APC, PE Armenian Hamster IgG Isotype Ctrl Antibody, PE Rat IgG2a,k Isotype Ctrl Antibody, APC Armenian Hamster IgG Isotype Ctrl Antibody, anti‐CD3‐PerCP‐Cy5.5, anti‐CD11b‐PE, anti‐F4/80‐APC, anti‐CD206‐FITC, anti‐iNOS‐FITC, anti‐CD4‐FITC, anti‐CD25‐PE and anti‐Foxp3‐APC, detail showed in Table 1. The monoclonal antibodies used above were either rat antimouse or rabbit antimouse. BD FACSCanto‐II (BD Bioscience) was used for acquisition, and the FlowJo software was used to analyse the outcome.

**Table 1:** Antibodies used for flow cytometry

Reagent	Source	Catalog#
PE antimouse CD11c antibody	BioLegend	117307
PE Armenian Hamster IgG Isotype Ctrl Antibody	BioLegend	400907
PE antimouse Ly‐6A/E(Sca‐1) antibody	BioLegend	108107
PE antimouse/human CD45R/B220 antibody	BioLegend	103207
PE antimouse CD140a(PDGFR‐a) antibody	BioLegend	135905
PE Rat IgG2a,k isotype Ctrl antibody	BioLegend	400507
CD29 monoclonal antibody, APC	eBioscience	17‐0291‐80
Armenian Hamster IgG Isotype Ctrl, APC	eBioscience	17‐4888‐82
CD3e monoclonal antibody, PerCP‐Cy5.5	eBioscience	45‐0031‐80
PE antimouse/human CD11b antibody	BioLegend	101207
APC antimouse F4/80 antibody	BioLegend	123115
FITC antimouse CD206(MMR) antibody	BioLegend	141703
iNOS monoclonal antibody, FITC	eBioscience	53‐5920‐82
CD4 monoclonal antibody, FITC	eBioscience	11‐0041‐82
CD25 monoclonal antibody, PE	eBioscience	12‐0251‐82
FOXP3 monoclonal antibody, APC	eBioscience	17‐5773‐82

### 2.7 Real‐time PCR

The RNA level expression of TGF‐β1 was examined by real‐time PCR. Firstly, total RNA was extracted using TRIzol (Takara). The extracted RNA with the OD260/OD280 nm absorption ratio range from 1.8 to 2.0 was reverse transcribed into cDNA using the PrimeScript RT Master Mix kit (Takara). The transcribed cDNA was used as template for real‐time quantitative PCR on a ViiA™7 Real‐Time PCR System (Applied Biosystems). Primers were obtained from Sangon Biotech. The following gene‐specific primers for real‐time PCR were used: TGF‐β1 (forward), 5′‐AGCTGCGCTTGCAGAGATTA‐3′ and TGF‐β1 (reverse), 5′‐CAGCCACTCAGGCGTATCAG‐3′; GAPDH (forward), 5′‐GTCAACGGATTTGGTCTGTATT‐3′ and GAPDH (reverse), 5′‐AGTCTTCTGGG TGGCAGTGAT‐3′. Real‐time PCR was carried out in triplicate. For quantification, we calculated the relative mRNA expression levels of TGF‐β1 according to the 2^−ΔΔCT^ method and normalized with GAPDH housekeeping gene.

### 2.8 Western blot

Western blot assays were carried out to detect the translation efficiency of TGF‐β1 in MSC. MSC, MSC transduced with LV‐GFP‐puro (MSC‐GFP‐puro) and LV‐TGF‐β1 (MSC‐TGF‐β1) were washed with PBS before lysis with RIPA buffer (Solarbio) containing 0.01% protease inhibitor cocktail (Sigma‐Aldrich) for protein extraction. The protein concentration was quantified using the BCA protein assay kit (Beyotime). Equal levels of protein were loaded in 10% SDS‐polyacrylamide gel electrophoresis and then transferred to polyvinylidenedifloride (PVDF) membranes (Millipore). After blocking with 5% milk for 2 hours, the membranes were incubated with primary antibody against DYKDDDK Tag (1:1000; CST) and TGF‐β1 (1:1000; CST) and GAPDH (1:1000; Beyotime) overnight at 4°C. This was followed by incubation with HRP‐conjugated secondary antibody (Beyotime Biotechnology) at room temperature for 2 hours. The detection was performed by ECL Chemiluminescence Detection Kit (Thermo Scientific). All experiments were performed in triplicate.

### 2.9 Co‐culture experiments

Mesenchymal stem cell, MSC‐GFP‐puro and MSC‐TGF‐β1 were seeded into 24‐well plates (Corning) with a density of 5 × 10^4^/well and incubated in 0.5 mL complete RPMI 1640 medium overnight. Splenocytes were extracted from Balb/c mice (6w). Mouse 1× Lymphocyte Separation Medium (Dakewe) was used to obtain splenic lymphocytes according to the manufacturers' instructions. The separated lymphocytes were stained with 1 mmol/L of carboxyfluorescein diacetate succinimidyl ester (CFSE, eBioscience) before co‐culture and then seeded into the well with MSC, MSC‐GFP‐puro and MSC‐TGF‐β1 at a density of 5 × 10^5^/well. The previous medium was changed into 0.5 mL fresh RPMI 1640 culture medium containing 10% FBS, 2 mmol/L glutamine, 100 U/mL penicillin and streptomycin, and 2 μg/mL ConA (Sigma). Lymphocytes labelled with CFSE cultured alone with or without ConA served as controls.

### 2.10 T lymphocytes proliferation assay

After the cells were co‐cultured for 3 days, suspension cells were harvested and labelled with anti‐CD3‐PerCP‐Cy5.5 (eBioscience) as a maker of T cells. The proliferation of CD3^+^T cells was analysed by visualizing CFSE fluorescence with a BD FACSCanto‐II flow cytometer.

### 2.11 ELISA

The level of IFN‐γ in the supernatant or in the serum was measured by IFN‐γ Quantikine ELISA Kit (eBioscience) according to the manufactures' protocols.

### 2.12 Mouse aGVHD model

The protocol of establishing mouse aGVHD model was according to previously published method.^25^ 5‐7 recipient mice each group for prophylactic experiments, 5 recipient mice each group for therapeutic experiments. Recipient (BALB/c) mice were fed water containing gentamicin sulphate (32 × 10^4^ U/L) and erythromycin (250 mg/L) 7 days before transplantation to prevent intestinal infection. On the day of transplantation, recipient BALB/c mice (female, 18‐20 g) received total body irradiation (TBI) with a myeloablative dose of 8.5 Gy followed by tail intravenous infusion of BMC (5 × 10^6^) and splenocytes (SC, 1 × 10^7^) extracted from C57BL/6 (male, 6‐8 weeks) mice. At the same time, recipient mice in the prophylactic experiments were, respectively, injected with MSC (1 × 10^6^), MSC‐GFP‐puro (1 × 10^6^) or MSC‐TGF‐β1 (1 × 10^6^) simultaneously through tail vain according to the groups they were assigned to. Recipient mice in the therapeutic experiments were injected with the first dose of MSC‐GFP‐puro (3 × 10^5^) or MSC‐TGF‐β1 (3 × 10^5^) according to the groups they were assigned to, followed by additional weekly dose of 3 × 10^5^ (MSC‐GFP‐puro/MSC‐TGF‐β1) cells for 4 weeks until the mice died. The group only transplanted with BMC and SC served as control group.

### 2.13 Clinical and pathologic scoring of aGVHD

Clinical features of aGVHD were monitored every 4 days from day 1 to day 29 post‐transplantation based on five separate parameters: weight loss, posture, activity, fur texture and skin integrity, and the detailed description of the scoring system are shown in Table 2.^26^ Three weeks after transplantation, one mouse from each group on same day 21 after transplant was randomly killed, and histological tissue samples from aGVHD target organs (liver, lung, small intestine and skin) were assessed. The tissues were fixed in 10% formalin, embedded in paraffin, stained with haematoxylin and eosin (H&E) and scored for aGVHD by a pathologist blindly.

**Table 2:** Assessment of clinical GVHD in transplanted animals

Criteria	Grade 0	Grade 1	Grade 2
Weight loss	<10%	>10% to <25%	>25%
Posture	Normal	Hunching noted only at rest	Severe hunching impairs movement
Activity	Normal	Mild to moderately decreased	Stationary unless stimulated
Fur texture	Normal	Mild to moderate ruffling	Severe ruffling/poor grooming
Skin integrity	Normal	Scaling of paws/tail	Obvious areas of denuded skin

### 2.14 Extraction and analysis of macrophages

We isolated macrophages from the mice by peritoneal lavage with DMEM medium. Briefly, 5 mL of pre‐cooled DMEM medium was injected into the peritoneal cavity of mice, and the abdomen of mice was gently massaged for 2‐3 minutes. Peritoneal fluid was collected with a syringe avoiding damage to the blood vessels or organs to ensure the absence of contamination from these sources. The procedures above were repeated twice to obtain as many cells as possible. Finally, the harvest cells were analysed by flow cytometry. Antibody information is listed in Table 1.

### 2.15 Immunofluorescence staining

Liver and lung tissue specimens were immediately obtained when the mice were killed at day 21 and subsequently fixed in 4% paraformaldehyde for 24 hours at 4°C. Samples were embedded in optimal cutting temperature compound and then sectioned. The sections (4 μm) were incubated overnight at 4°C with primary antibodies against F4/80 (1:400, Abcam) and CD206 (1:400, Abcam) and then incubated with FITC‐conjugated (anti‐F4/80) or CY3‐conjugated (anti‐CD206) secondary antibody (1:500, Santa Cruz Biotechnology). After washing with PBS, the sections were mounted in fluorescent mounting medium containing 4′,6‐diamidino‐2‐phenylindole (DAPI) (Beyotime). The outcome was examined and photographed using fluorescence microscopy (Olympus BX51). Not less than three fields at ×100 magnification per section were randomly selected as representatives for analysis and calculation.

### 2.16 Statistical analysis

All statistical analyses were performed using SPSS 20.0 (SPSS Inc, Chicago, IL, USA), and graphs were generated using the GraphPad Prism software (version 5.0.1; La Jolla, CA). Data were expressed as mean values ± standard deviation. One‐way analysis of variance (ANOVA) was performed to assess the effects of a single factor on multiple groups. Tukey's multiple comparison test was used as post hoc test. Log‐rank test was used to assess the statistical survival differences. Animal sample size was calculated using sample size calculating software G*Power version 3.1.9.2 (Franz Faul, Universitaet Kiel, Germany) with a α‐value of .05, a power of 80%. All the *P*‐values are two‐sided, and *P* < .05 is taken as statistically significant.

## 3 RESULTS

### 3.1 Characterization of mice bone marrow‐derived MSC

**Figure 1:**
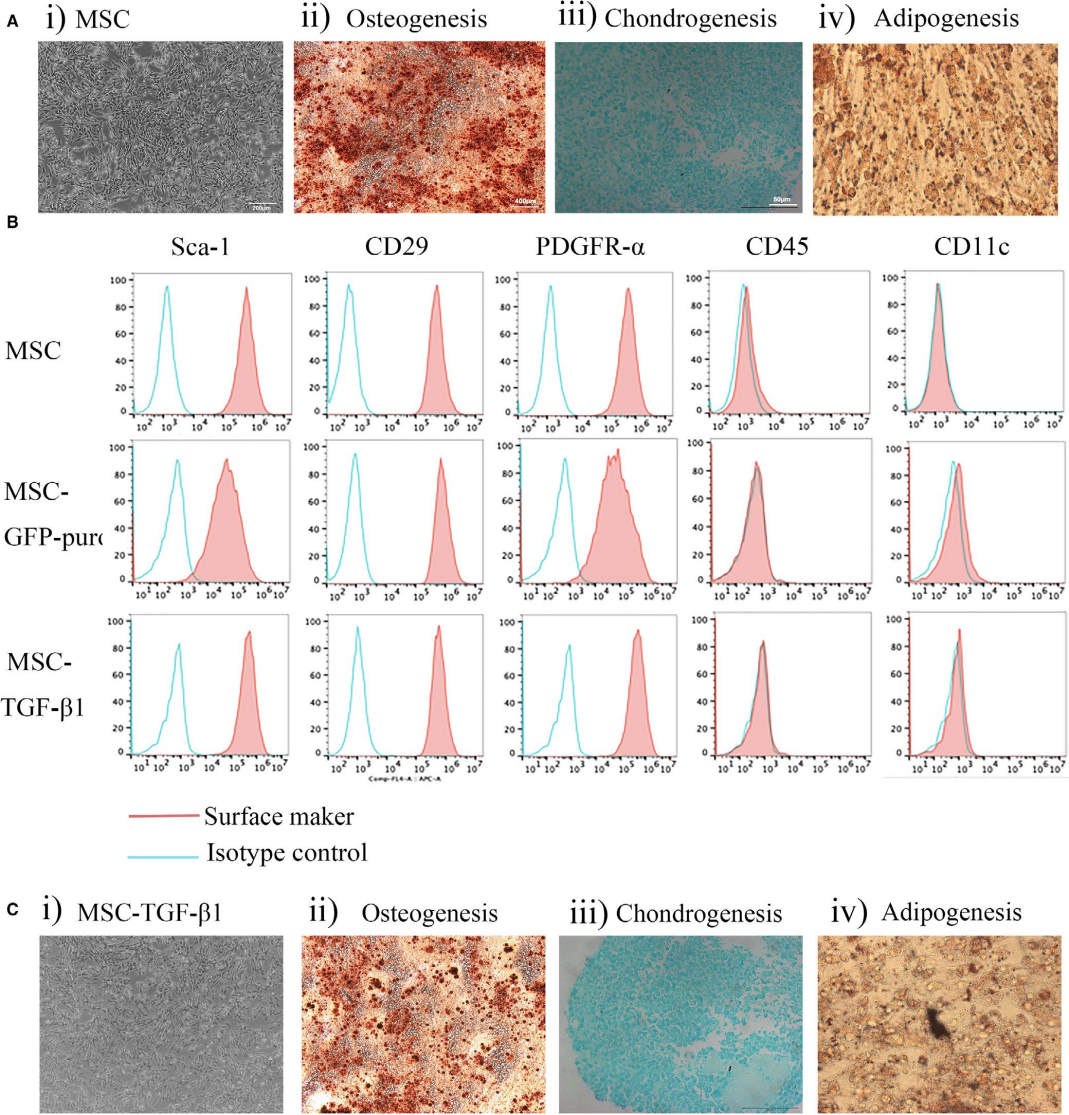
Mouse bone marrow mesenchymal stem cells (MSC) were isolated successfully. A, Mouse bone marrow‐derived MSCs showed morphology consistent with typical MSC (i) and could successfully differentiate to osteoblasts (ii), chondrocytes (iii) and adipocytes (iv). B, Surface markers of MSC, MSC‐GFP‐puro and MSC‐TGF‐β1 were analysed by flow cytometry. C, MSC‐TGF‐β1 morphology was similar to normal MSC (i) and could differentiate to osteoblasts (ii), chondrocytes (iii) and adipocytes (iv). Data are representative of three independent experiments for A‐C

Under inverted light microscopy, the cultured normal bone marrow‐derived murine MSCs were all plastic adherent long and polygonal cells with similar morphology to fibroblasts, as showed in Figure 1A,i. Moreover, MSC showed the ability to differentiate into osteogenic (Figure 1A,ii), chondrogenic (Figure 1A,iii) and adipogenic (Figure 1A,iv) lineages. Furthermore, MSCs presented typical MSC immunophenotype, with positive expression for CD29, Sca‐1, PDGFR‐α and negative for CD11c, CD45 (Figure 1B).

### 3.2 TGF‐β1 transduced MSC share same immunotypic features, morphology and differentiation ability of normal MSC

**Figure 2:**
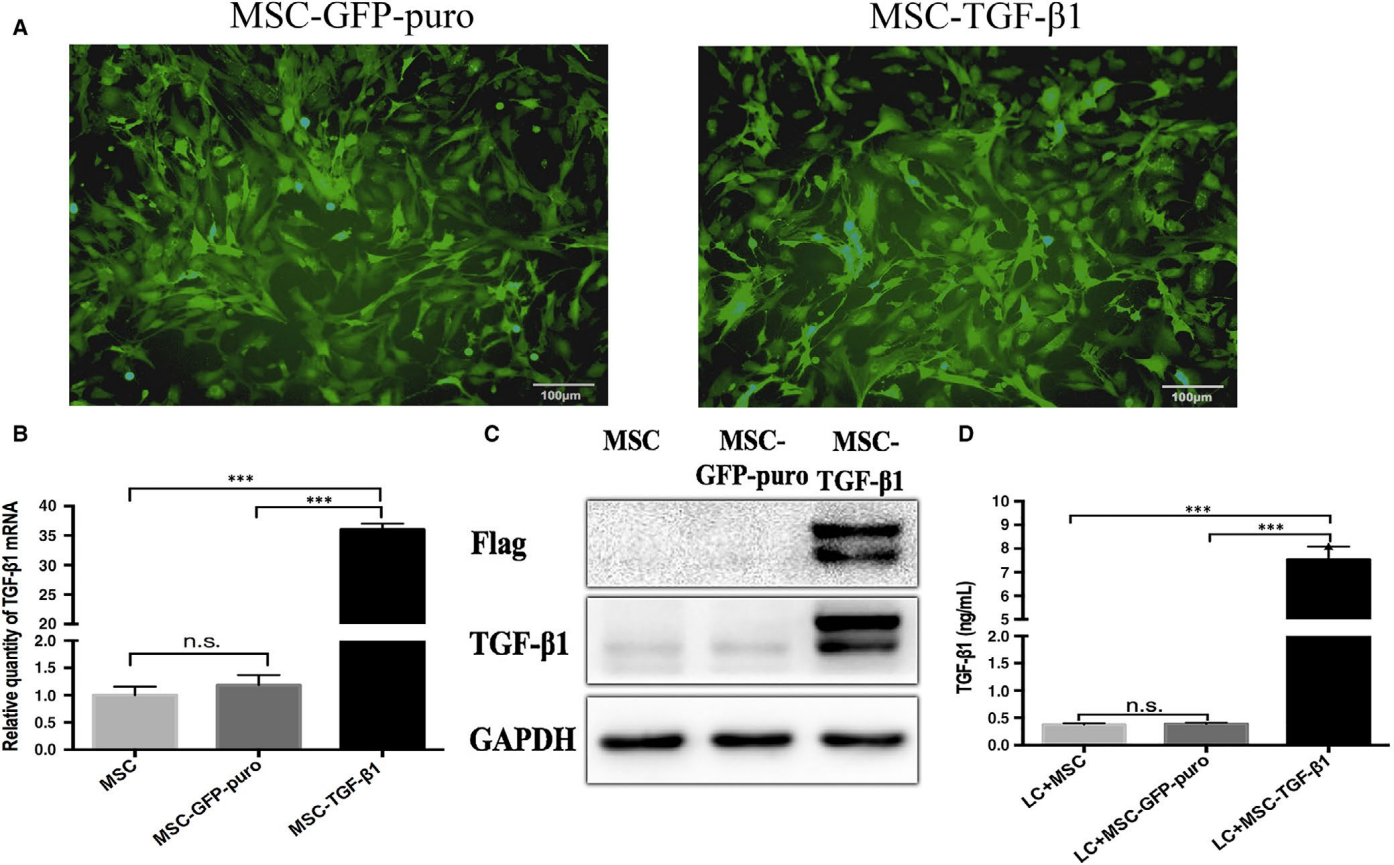
TGF‐β1 was successfully transduced into MSC by lentivirus transfection. MSC was transduced with lentivirus and then tested for the transduction efficiency. Supernatants from 1 × 10^5^ MSC/MSC‐GFP‐puro/MSC‐TGF‐β1 were collected at 24 h and assayed by ELISA kits for TGF‐β1. A, MSC transfected with LV‐GFP‐puro and LV‐TGF‐β1 was observed under fluorescence microscope. B, Relative expression of TGF‐β1 mRNA in MSC, MSC‐GFP‐puro and MSC‐TGF‐β1 by real‐time PCR. C, The translation levels of flag and TGF‐β1 protein by Western Blot. D, Levels of TGF‐β1 in the supernatants of MSC/MSC‐GFP‐puro/MSC‐TGF‐β1. Data are representative of three independent experiments for A‐B. Data are presented as the mean ± SD. ***P < .001, n.s. not significant

We evaluated the immunotypic biomarker of MSC‐GFP‐puro and MSC‐TGF‐β1 by flow cytometry. As shown in Figure 1B, these cells showed a typical MSC pattern: being strongly positive for CD29, Sca‐1, PDGFR‐α; and negative for CD11c, CD45. These findings indicated that LV‐GFP‐puro and LV‐TGF‐β1 transduction did not alter the normal morphology or immunotype of MSC. In addition, MSC‐TGF‐β1 also could differentiate into osteogenic (Figure 1C,ii), chondrogenic (Figure 1C,iii) and adipogenic (Figure 1C,iv) lineages. Finally, as normal MSC, MSC‐GFP‐puro and MSC‐TGF‐β1 were all plastic adherent long or polygonal cells as showed in Figure 2A. This laid a solid foundation for our following research.

### 3.3 TGF‐β1 successfully transduced MSC resulting in high expression

As GFP gene was present in both lentivirus HBLV‐m‐TGF‐β1‐3xflag‐GFP‐puro and HBLV‐GFP‐puro, the MSCs transduced with lentivirus produced high level of GFP under fluorescence microscopy, as shown in Figure 2A. To further confirm this, we examined the mRNA expression levels of TGF‐β1 by real‐time PCR in three groups of cells. As showed in Figure 2B, TGF‐β1 mRNA levels were the highest in MSC‐TGF‐β1 group compared with MSC group or MSC‐GFP‐puro group (*P* < .001). At the same time, translation efficacies of TGF‐β1 and flag protein were detected in three groups by Western Blot, which showed consistent results with the outcome of real‐time PCR where TGF‐β1 and flag protein concentrations were significantly increased in MSC‐TGF‐β1 (Figure 2C). Furthermore, we tested TGF‐β1 production in the supernatant of cultured cells and found that the MSC‐TGF‐β1 group was the highest (Figure 2D). Taken together, target gene TGF‐β1 was successfully transduced and ectopically overexpressed in MSC‐TGF‐β1.

### 3.4 TGF‐β1‐transduced MSC manifest enhanced immunosuppressive capacity on T lymphocyte in vitro

**Figure 3:**
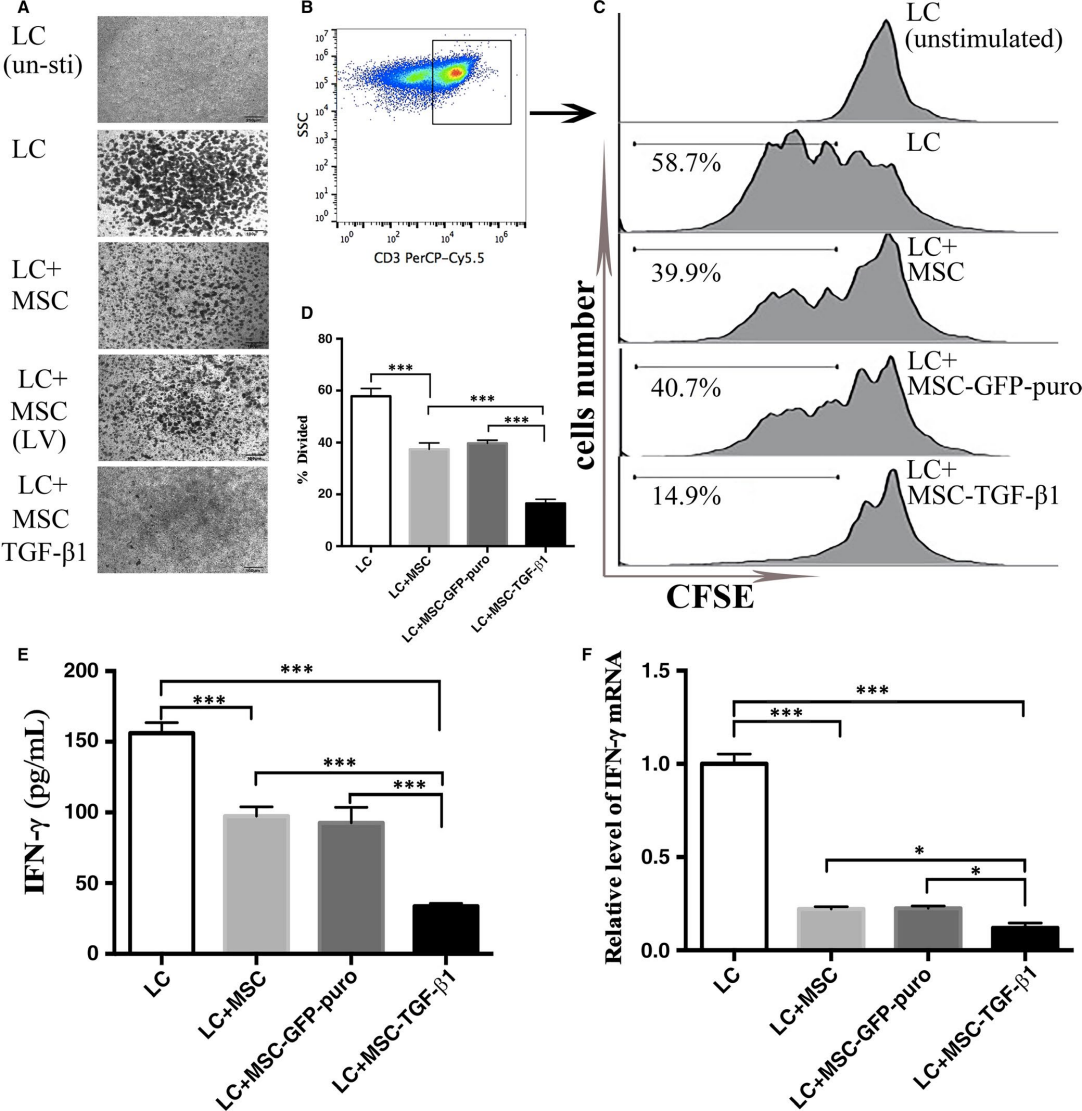
Immunosuppressive effect of MSC, MSC‐GFP‐puro and MSC‐TGF‐β1 on lymphocyte in vitro. Lymphocytes isolated from the spleens of C57 mice were pre‐labelled with CFSE and then co‐culture with MSCs/MSC‐GFP‐puro/MSC‐TGF‐β1 in 24‐well plates at a ratio of 10:1 for 3 d. Lymphocytes cultured alone supplemented with or without ConA were set as positive and negative controls. A, Inverted light microscopic appearance of lymphocyte colonies. B, Suspension cells in the culture system were collected and stained with anti‐CD3‐PerCP‐Cy5.5 for the flow cytometry analysis of CD3^+^T cells. C‐D, Proliferation of T cells in all groups as detected by CFSE assays and analysed by the FlowJo software. E, IFN‐γ concentration in the culture supernatant as measured by ELISA. F, Relative expression of IFN‐γ mRNA of the suspension cells as measured by real‐time PCR. Data are representative of three independent experiments for A‐E. Data are presented as the mean ± SD. *P < .05, ***P < .001

To investigate whether MSC‐TGF‐β1 possess enhanced suppressive function on T lymphocytes, we firstly isolated normal lymphocytes from the spleen of Balb/c mice, and then, we co‐cultured MSC/MSC‐GFP‐puro/MSC‐TGF‐β1 (5 × 10^4^), respectively, with the isolated lymphocytes (5 × 10^5^) which were pre‐labelled with CFSE at the ratio of 1:10. The co‐culture system was supplemented with additional ConA, which acts as immunostimulant for mice lymphocytes. Cells were incubated at 37°C in a humid atmosphere of 5% CO_2_ for 3 days. When observed under inverted light microscope (Figure 3A), lymphocytes cultured alone without ConA did not show any colony formation, while lymphocytes cultured with ConA formed large numbers of colonies. The groups where lymphocytes were co‐cultured with MSC or MSC‐GFP‐puro both showed a reduction in lymphocyte colonies. The MSC‐TGF‐β1 co‐cultured group showed the fewest lymphocyte colonies. Suspension cells were collected and stained with anti‐CD3‐PerCP‐Cy5.5 for flow cytometry. CFSE assays showed that MSC, MSC‐GFP‐puro, MSC‐TGF‐β1 all suppressed the proliferation of activated CD3^+^ T cells compared with control group where lymphocytes cultured alone (Figure 3B‐D). Of note, MSC‐TGF‐β1 exerted the most marked suppression on lymphocytes proliferation. Mean value ± SD of proliferation rate for LC, MSC, MSC‐GFP‐puro and MSC‐TGF‐β1 was 57.90 ± 2.90, 37.38 ± 2.49, 39.71 ± 1.13, 16.6 ± 1.47, respectively. In addition, we found that IFN‐γ secretion was markedly decreased in the culture medium of MSC‐TGF‐β1 group by ELISA method (Figure 3E), which were supported by detecting the RNA quantity in the cultured lymphocytes using real‐time PCR (Figure 3F).

### 3.5 Prophylactic application of TGF‐β1‐transduced MSC markedly ameliorated the severity of aGVHD and improved overall survival in murine model

**Figure 4:**
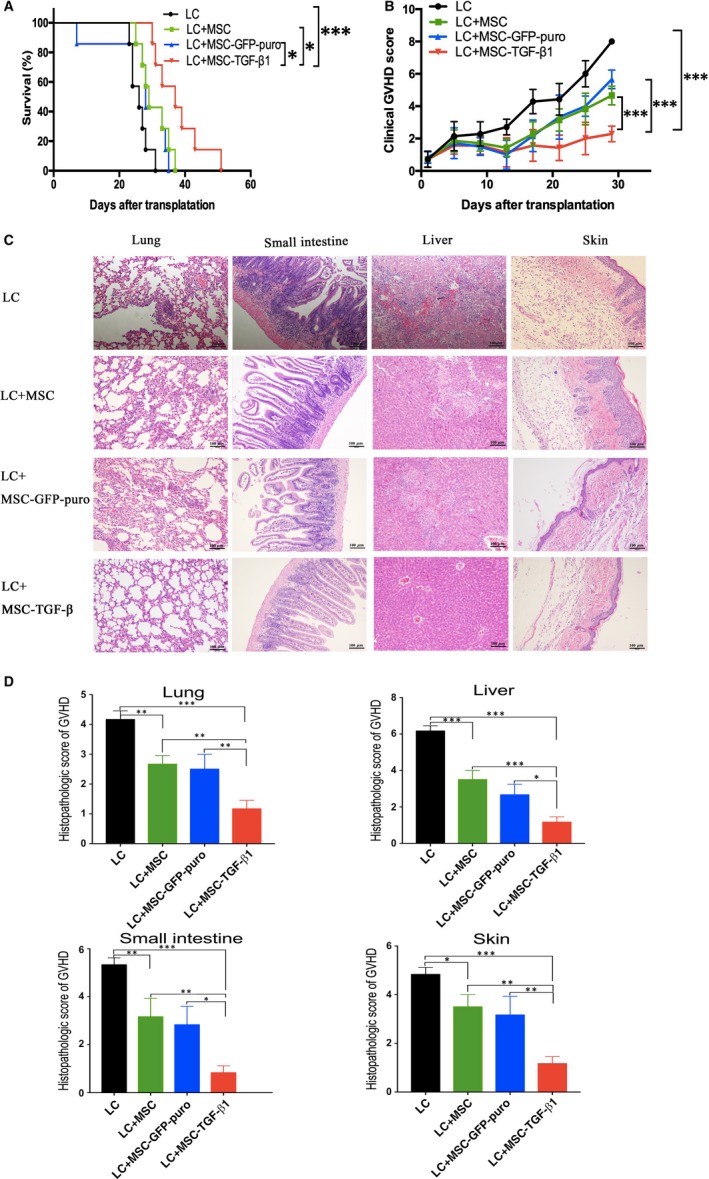
Prophylactic application of MSC‐TGF‐β1 ameliorated the severity of aGVHD in mice. After total body irradiation with a myeloablative dose of 8.5 Gy, the recipient mice were transplanted with donor murine bone marrow cells (BMC: 5 × 10^6^) and spleen cells (SC: 1 × 10^7^), with or without MSC/MSC‐GFP‐puro/MSC‐TGF‐β1 (1 × 10^6^) according to the groups they were assigned to. A, Overall survival of mice in each group. B, Clinical GVHD score of mice in each group. C, Representative histopathology images of lung, small intestine, liver and skin tissue on day 21 after transplantation. D, Histopathology scores for samples of lung, small intestine, liver and skin on day 21 after transplantation. Data are representative of three independent experiments with 5‐7 mice per group. Data are presented as the mean ± SD. *P < .05, **P < .01, ***P < .001

Mice were allocated to four groups intravenously injected with BMC+SC, BMC+SC+MSC, BMC+SC+MSC‐GFP‐puro or BMC+SC+MSC‐TGF‐β1. Considering that mice of BMC+SC group all died within 31 days post‐transplant, we performed aGVHD clinical scores on each group of mice every 4 days only from day 1 to day 29 after transplant. Mice infused with only BMC (5 × 10^6^) and SC (1 × 10^7^) in a ratio of 1:2 gradually developed symptoms of severe aGVHD at about 17 days after transplantation, with a median survival of 26 days. The mice infused with BMC, SC and MSC/MSC‐GFP‐puro/MSC‐TGF‐β1 (1 × 10^6^) showed prolonged overall survival and improvement in aGVHD severity to variable degrees (Figure 4A,B). Mean value ± SD of clinical score in Figure 4B for BMC+SC, BMC+SC+MSC, BMC+SC+MSC‐GFP‐puro and BMC+SC+MSC‐TGF‐β1 groups was 29.71 ± 3.30, 24.71 ± 5.82, 24 ± 3.11 and 12.28 ± 3.77, respectively. In particular, mice treated with MSC‐TGF‐β1 developed the mildest aGVHD manifestation and correspondingly had the longest median survival of 37 days. Although infusion of MSC/MSC‐GFP‐puro could delay the onset of GVHD, the severity of the disease was not ameliorated. Mice treated with MSCs and MSC‐GFP‐puro developed aGVHD of similar clinical scores and had comparable survival, indicating that empty vector lentiviral transfection did not enhance the immunosuppressive effect of MSCs. In our preliminary research, we found that most mice manifested onset of aGVHD from day 21 when the donor cells were fully engrafted. On the contrary, mice in each group gradually died 4 weeks post‐transplant due to aGVHD. So, it was theoretically unable to find discrepancy between different groups too early or too late post‐transplant. Though it could give a full view of the pathological difference, we could not analyse overall survival of mice if we killed all mice simultaneously. To compromise this problem, we randomly killed one mouse from each group on same day 21 after transplant. Totally, histopathology of 3 mice for each group from 3 independent experiments was statistically analysed. We performed pathological scoring on mouse organ according to the aGVHD pathological scoring criteria,^27^ and compared the severity of aGVHD. We found that pathological inflammatory changes in all the target organs were reduced to a variable degree in the 3 groups of mice transplanted with MSC/MSC‐GFP‐puro/MSC‐TGF‐β1. Specifically, the tissue damage was mildest in the group treated with MSC‐TGF‐β1 (Figure 4C,D), suggesting that prophylactic application of MSC‐TGF‐β1 ameliorated the severity of aGVHD and improved the survival of this murine model.

### 3.6 MSC‐TGF‐β1 promoted the M2 macrophage polarization in aGVHD mice

**Figure 5:**
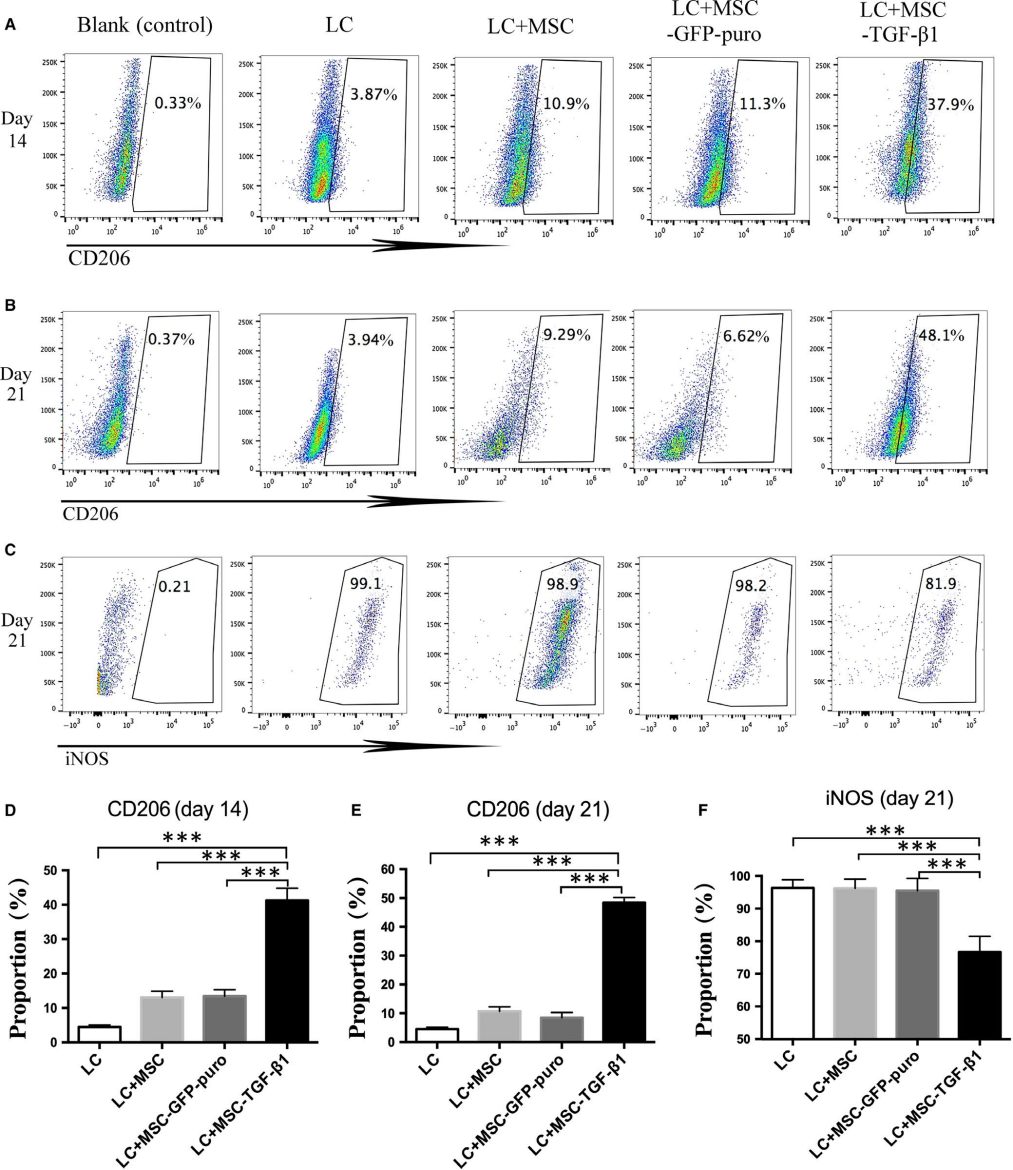
MSC‐TGF‐β1 altered the phenotypes of mice peritoneal macrophages. Macrophages were harvested from mice peritoneal cavity and analysed by flow cytometry on their expression of certain makers. A, CD206 expression in macrophages at day 14 after transplantation B, CD206 expression in macrophages at day 21 after transplantation. C, iNOS expression in macrophages at day 14 after transplantation. D, Quantitative analysis of the expression of CD206 in macrophages at day 14 after transplantation. E, Quantitative analysis of the expression of CD206 in macrophages at day 21 after transplantation. F, Quantitative analysis of the expression of iNOS in macrophages at day 21 after transplantation. Data are representative of three independent experiments with 5‐7 mice per group. Data are presented as the mean ± SD. ***P < .001

**Figure 6:**
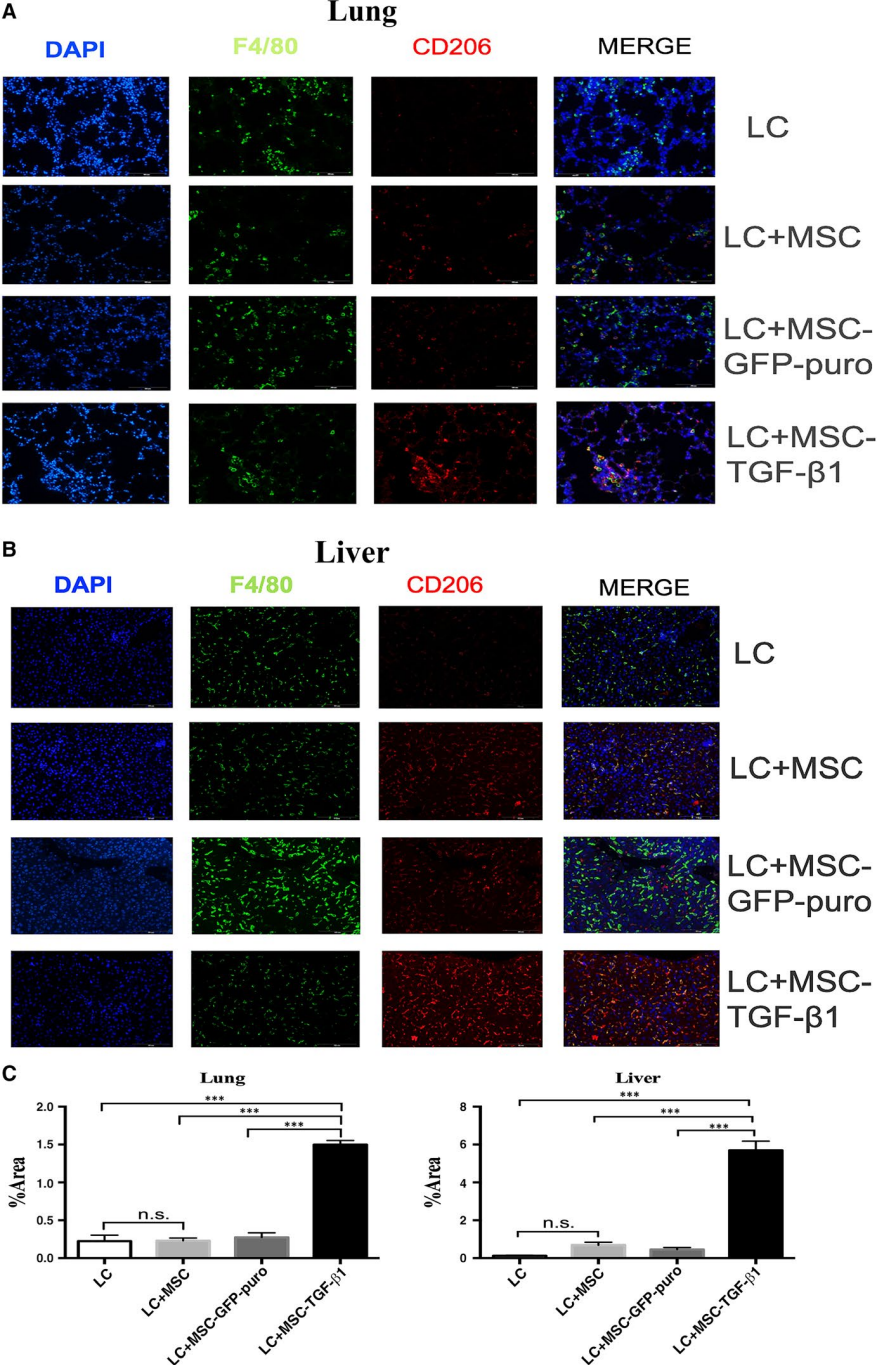
MSC‐TGF‐β1 enhanced the infiltration of M2 macrophage in lung and liver tissues of mice. Lung and liver tissues of killed mice were collected and detected the infiltration of M2 macrophage through CD206 staining by immunofluorescence analysis. DAPI staining was used to indicate overall cell nucleus, and cell surface marker staining of F4/80 was used to indicate macrophages, CD206 staining represent M2 macrophage. A, CD206 protein expression in lung. B, CD206 protein expression in liver. C, Quantification of CD206 protein expression in lung by densitometry analyses. D, Quantification of CD206 protein expression in liver by densitometry analyses. *** *P*＜.001

To clarify its underlying mechanism of how MSC‐TGF‐β1 ameliorates the severity of aGVHD and improves survival of murine model, we investigated the effect of MSC on phenotypes of the macrophages, a vital immunoregulatory cell type. Two major macrophage subpopulations with different functions including classically activated/inflammatory (M1) and alternatively activated/regenerative (M2) macrophages have long been recognized. We killed mice of all groups at day 14 and 21 after transplantation and harvested macrophages from the peritoneal cavity using the methods described.^28^ Flow cytometry was used to detect the proportion of CD11b^+^ F4/80^+^ CD206^+^ M2‐like macrophages and CD11b^+^ F4/80^+^ iNOS^+^ M1‐like macrophages in total macrophages (CD11b^+^ F4/80^+^ macrophage). Compared with mice transplanted with BMC and SC, the M2‐like macrophage proportion in mice receiving additional MSC, MSC‐GFP‐puro or MSC‐TGF‐β1 were significantly increased on 14th and 21st days after transplantation (Figure 5A,B,D,E). Mean value ± SD of CD206 proportion in Figure 5D for BMC+SC, BMC+SC+MSC, BMC+SC+MSC‐GFP‐puro and BMC+SC+MSC‐TGF‐β1 groups was 4.48 ± 0.57, 13 ± 1.84, 13.4 ± 1.87 and 41.27 ± 3.56, respectively. Interestingly, the percentage of M2‐like macrophages was highest in the MSC‐TGF‐β1 group. Furthermore, we found that at 21 days after transplantation, the expression of iNOS by macrophages (M1‐like) was lowest in MSC‐TGF‐β1 group (Figure 5C,F). These results indicate that the infusion of MSC‐TGF‐β1 enhanced M2 macrophage polarization in aGVHD mice. Furthermore, we analysed M2 macrophage in lungs and liver by immunofluorescence in Figure 6. Coincided with the results of flow cytometry analysis of macrophage from peritoneal cavity, CD206 protein expression of lung (Figure 6A,C) and liver (Figure 6B,D) sample was the highest in MSC‐TGF‐β1–treated groups.

### 3.7 MSC‐TGF‐β1 increased the proportion of Treg cells in peripheral blood of aGVHD mice

**Figure 7:**
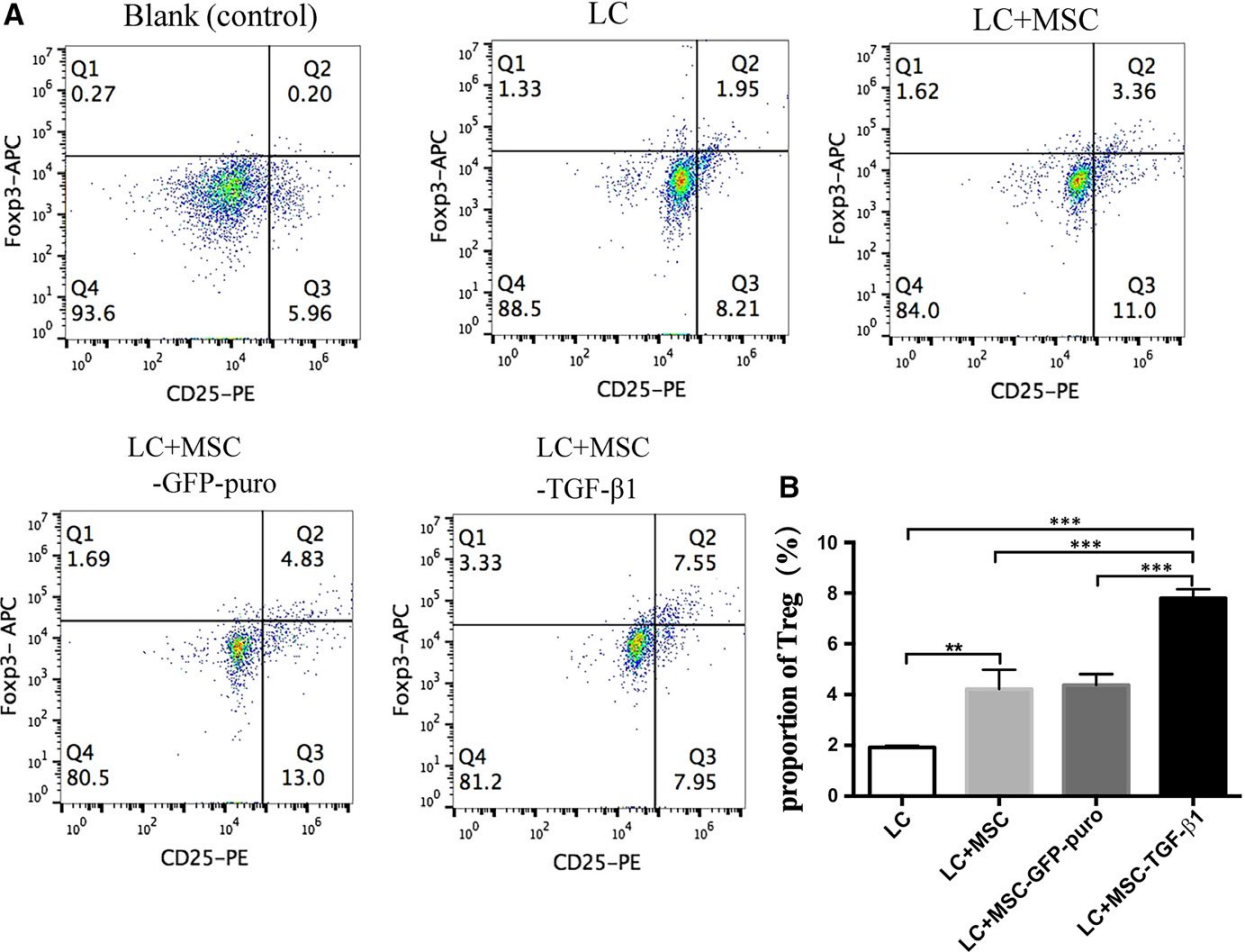
MSC‐TGF‐β1 increased the proportion of Foxp3^+^ Treg cells. Mononuclear cells were extracted from mice blood and analysed on day 21 post‐transplant by flow cytometry on the proportion of Foxp3^+^ Treg cells. A, Proportion of Foxp3^+^ Treg cells in CD4^+^ T cells. B, Quantitative analysis of the proportion of Foxp3^+^ Treg cells. Data are representative of three independent experiments with 5‐7 mice per group. Data are presented as the mean ± SD. *P < .05, **P < .01, ***P < .001

To further investigate other possible mechanism of MSC‐TGF‐β1 exert effect on murine aGVHD model, we analysed the mononuclear cells from blood of mice at day 21 for detection of CD4^+^CD25^+^FoxP3^+^ Treg cells by flow cytometry. We found that the proportion of Treg cells in MSC‐TGF‐β1–infused mice was much higher than other groups, with the proportion 1.95% for LC group, 3.36% for MSC group, 4.83% for MSC‐GFP‐puro group and 7.75% for MSC‐TGF‐β1 group, respectively (Figure 7A,B). Mean value ± SD of Treg proportion in Figure 7B for BMC+SC, BMC+SC+MSC, BMC+SC+MSC‐GFP‐puro and BMC+SC+MSC‐TGF‐β1 groups was 1.92 ± 0.07, 4.22 ± 0.76, 4.38 ± 0.43 and 7.82 ± 0.34, respectively. This indicated that TGF‐β1‐transduced MSC ameliorated the severity of aGVHD partly through its effect on Treg cells.

### 3.8 Therapeutic application of TGF‐β1‐transduced MSC alleviated the clinical symptoms and improved overall survival in murine model of aGVHD

**Figure 8:**
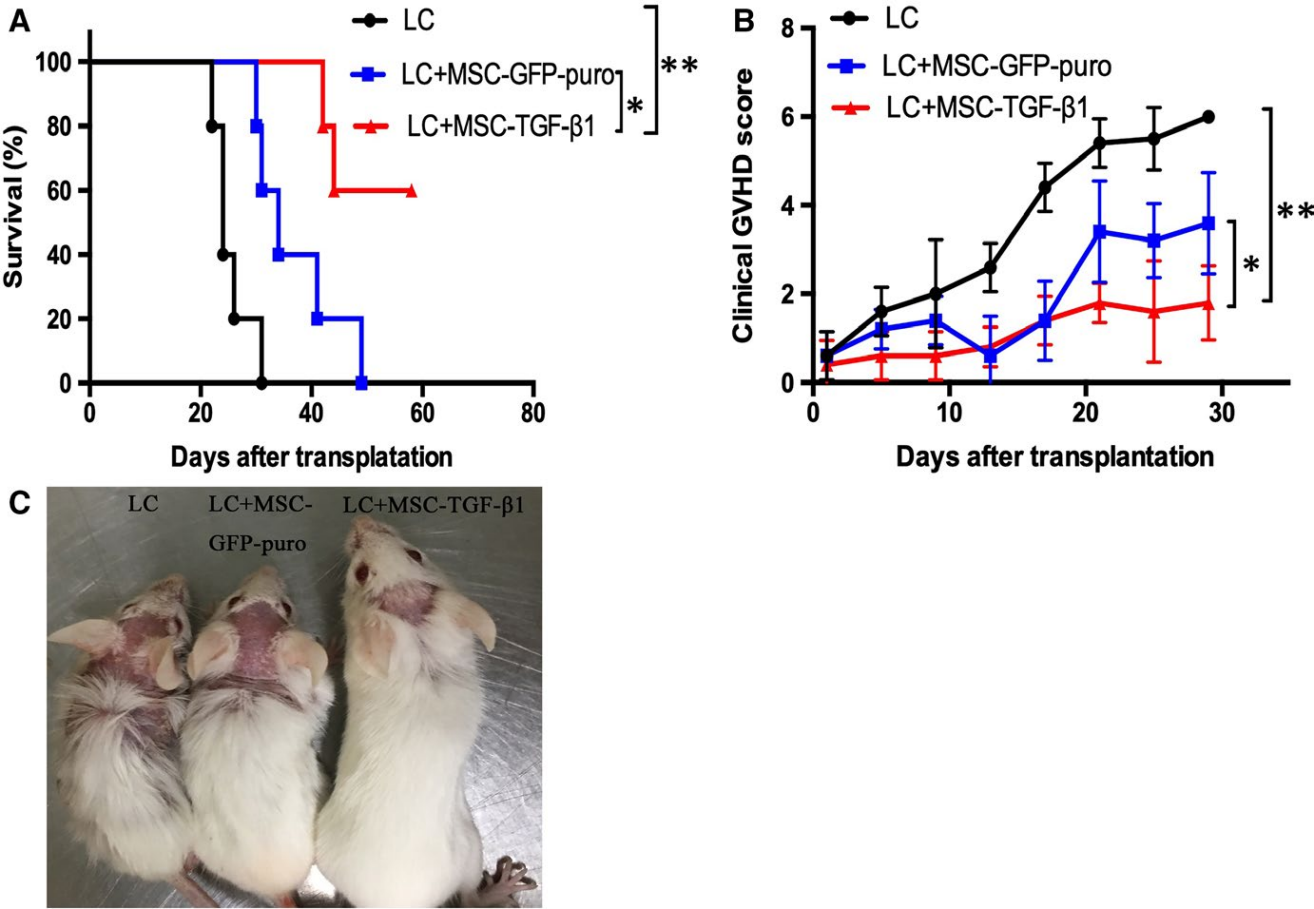
Therapeutic application of multiple doses MSC‐TGF‐β1 showed advantage in treating aGVHD. After total body irradiation with a myeloablative dose of 8.5 Gy, the recipient mice were transplanted with donor murine bone marrow cells (BMC: 5 × 10^6^) and spleen cells (SC: 1 × 10^6^) in the LC group. And in other groups, MSC‐GFP‐puro or MSC‐TGF‐β1 (3 × 10^6^) was infused to recipient mice for the first dose and followed by additional weekly dose of 3 × 10^5^ cells for 4 weeks. A, Overall survival of mice in each group. B, Clinical aGVHD score of mice in each group. C, Graphic performance of mice (day 21) in each group. Data are representative of three independent experiments with 5 mice per group. Data are presented as the mean ± SD. *P < .05, **P < .01, ***P < .001

In order to observe the therapeutic effect of MSC‐TGF‐β1 on murine aGVHD model, mice were treated with weekly injection of MSC‐GFP‐puro (3 × 10^5^) or MSC‐TGF‐β1 (3 × 10^5^) after allo‐HSCT for consecutive 5 doses. As showed in Figure 8A, compared with the non‐treatment control, the median survival of mice in MSC‐GFP‐puro group was 34 days. Particularly, the MSC‐TGF‐β1 (3 × 10^5^)–treated group showed the best outcome with sixty per cent of mice achieved long‐term survival (more than 50 days post‐transplantation). Notably, the survival outcome of MSC‐TGF‐β1 therapeutic group was even superior to that of MSC‐TGF‐β1 prophylactic group mentioned in Figure 4A. Correspondingly, the clinical symptoms and aGVHD score in MSC‐TGF‐β1 group were the slightest (Figure 8B,C). Mean value ± SD of clinical score in Figure 8B for BMC+SC, BMC+SC+MSC‐GFP‐puro and BMC+SC+MSC‐TGF‐β1 groups was 28 ± 3.74, 15.4 ± 3.78 and 9 ± 1.58, respectively.

## 4 DISCUSSION

Coupling its immunomodulatory property with its ability of homing to damaged tissue to regulate inflammation and promote repair, MSC‐based treatment shows great promise in aGVHD. While several clinical trials have demonstrated efficacy of MSC in steroid‐refractory aGVHD,^13^, ^17^, ^29^ there are studies reporting to the contrary.^16^ The heterogeneity of the MSC population may be one reason for the inconsistent outcome,^30^ which underscores the importance of maintaining and further enhancing its immunoregulatory activity.

In the present study, we aimed to improve MSC‐based immunotherapy through lentivirus transduction of TGF‐β1 in MSC. Our work showed that MSC‐TGF‐β1 exhibited enhanced immunosuppressive functions in vitro. Compared with MSC and MSC‐GFP‐puro, MSC‐TGF‐β1 was most efficient in suppressing lymphocyte colony formation and T cell proliferation. These were consistent with the findings of lowest IFN‐γ concentration in the co‐culture supernatant of the MSC‐TGF‐β1 group. Mesenchymal stem cell can regulate immunity both by secreting soluble factors and by influencing the biology of immune cells.^9^ As a well‐characterized immunosuppressive factor, TGF‐β1 suppresses immune response either by inhibiting the function of immune cells or by altering their differentiation.^20^, ^21^, ^31^ It was documented that TGF‐β1 plays a crucial part in the immunoregulatory function of MSC.^32^, ^33^ In our study, TGF‐β1 gene‐modified MSC exerted powerful immunosuppression, which could be attributed to the synergistic effect of these two factors, consistent with a previous study.^34^ Another study held that TGF‐β1 abolished MSC‐mediated immunosuppressive effect on anti‐CD3–activated splenocytes,^35^ but in this study, recombinant TGF‐β1 was directly added to the culture media, in contrast to our method where TGF‐β1 was overexpressed in MSC by genetic engineering strategy.

In our animal studies, overexpressing TGF‐β1 improved the efficacy of MSC in treating aGVHD, as reflected by lower aGVHD clinical scores, attenuated histological evidence of aGVHD and an improved survival. aGVHD is a systemic disease involving many organs such as small intestine, liver, lung and skin.^36^ MSC owns the ability of homing to the damaged tissues, relieving the severity of inflammation and accelerating tissue repair.^37^ After intravenous injection, MSC‐TGF‐β1 may migrate to the damaged target organs and secrete TGF‐β1 at the site of lesion. Genetically engineered MSC, by overexpressing TGF‐β1, has been investigated in treating many autoimmune and inflammatory diseases, by inducing tolerance, facilitating regeneration and repair, and has been proven to be efficient and safe.^38^, ^39^ In this study, we proved that MSC‐TGF‐β1 was superior to MSC in delaying the onset of aGVHD and reducing the severity of aGVHD in mice, thereby offering a novel strategy for treating aGVHD. It must be mentioned here that mice in not only MSC and MSC‐GFP‐puro infused groups, but also MSC‐TGF‐β1–infused group succumbed to aGVHD eventually. It is reported that higher proportion of ‘ready to differentiate—MSCs’ with limited proliferative capacity amongst the transplanted MSCs, entrapment of transplanted MSCs in the reticuloendothelial system, fusion of transplanted MSCs with recipient's cells and in vivo differentiation of transplanted MSCs into non‐immunosuppressive cells appears to be some of the factors responsible for gradual decline in the number of ‘immunosuppressive—MSCs’.^18^, ^40^ As the infused MSC is cleared up from the body of mice, their immunomodulatory effect gradually weakens and eventually disappears. Clinical results also showed that multiple infusion of MSC achieved better treatment outcome on aGVHD than single infusion.^41^ To overcome this problem, we further explored multiple dosing intravenous administration of MSC‐TGF‐β1 to treat aGVHD mice in attempt to alleviate symptoms and further prolong survival. Preliminary studies have showed, compared with MSC‐GFP‐puro–treated group; it can greatly ameliorate the clinical symptoms of aGVHD and improve survival of aGVHD mice.

Depending on the microenvironment they are exposed to, macrophages exhibit versatility in phenotype and function.^42^ Typically, macrophages are categorized into two main groups, classically activated macrophages (M1) and alternatively activated macrophages (M2). The M1‐like macrophages mediate pro‐inflammatory effects, characterized by up‐regulated expression of inducible nitric oxide (iNOS). Conversely, the M2‐like macrophages are immunosuppressive cells characterized by the expression of CD206 and arginase 1 (Arg1).^43^ Previous research showed that MØs play an important role in the pathogenesis of aGVHD through producing TNF‐α after exposure to LPS,^44^ and human skin lesions infiltrated by CD163+ macrophage were proposed as a predictive factor for refractory GVHD associated with poor overall survival.^45^ In contrast, M2‐like macrophages reduced the inflammatory response.^46^ Considering that different subsets of macrophage play different roles in inflammation process, while GVHD is caused by donor T cell–mediated damage to recipient target organs either directly through cytolytic attack or indirectly through the release of inflammatory mediators. We postulated that macrophages might be involved in the underlying anti‐GVHD mechanism of MSC‐TGF‐β1. We found that macrophages derived from mice treated with MSC‐TGF‐β1 showed the highest expression of the M2 marker CD206, and a reduced expression of M1 marker iNOS. Three broad pathways control macrophage polarization: epigenetic and cell survival pathways that prolong or shorten macrophage development and viability, the tissue microenvironment, and extrinsic factors, such as microbial products and cytokines released in inflammation. Mesenchymal stem cell has been described to shift macrophages to M2‐like subsets,^47^, ^48^ and MSC‐educated macrophages possess the therapeutic potential for GVHD.^48^ Furthermore, as demonstrated previously, TGF‐β1 can increase the differentiation of M2‐like macrophages which function in wound healing and immunoregulation.^49^, ^50^ Taken together, these studies partially explain why infusion of MSC‐TGF‐β1 increased the M2‐like macrophages in our murine aGVHD models. However, the precise underlying mechanism how M2 macrophage polarizated and functioned is quite sophisticated and still unknown in our current study, which need further illustration. MSC‐TGF‐β1 might promote the generation of M2‐like macrophages either directly or through its effect on the immune environment. The increased M2‐like macrophages can regulate immune response probably by secreting proteins, such as IL‐10 and arginase 1 (Arg1).^51^ We speculate that these biological behaviours of M2‐like macrophages may play an important role in moderating the over‐active immune response in aGVHD. Lastly, we also found that MSC‐TGF‐β1–treated mice showed an increased in the proportion of Treg as compared to other groups. As we know, TGF‐β signalling plays a vital role in the development of natural CD4+CD25+Foxp3+ regulatory T cells.^52^ The exact mechanism through which Tregs control immune responses has not been fully elucidated. Treg function appears to be cytokine or contact mediated. Several studies showed that IL‐10, TGF‐β and IL‐35 have been implicated in enhancing suppression, whereas CTLA‐4, LAG‐3, CD39 and granzymes play an important role in the contact‐dependent immune control. In a number of different allogeneic HCT animal models, the addition of highly purified CD4+CD25+FoxP3+ Tregs resulted in suppression of GVHD.^53^ We hereby speculate that increased Treg cells might be involved in regulating immune response in aGVHD mice infused with MSC‐TGF‐β1.

It has been reported that multiple mechanisms are involved in immunosuppressive capacity of MSC in vivo. Antonio Galleu et al^54^ proved that not only cytotoxic cells in the recipient are required to initiate apoptosis in infused MSCs but also phagocytes that, by engulfing apoptotic MSCs and producing IDO, ultimately deliver MSC immunosuppressive activity. Another group^55^ demonstrated that infused MSC is rapidly phagocytosed by monocytes, which subsequently migrate from the lungs to other body sites. Phagocytosis of umbilical cord‐derived MSCs (UC‐MSCs) induces phenotypical and functional changes in monocytes, which subsequently modulate cells of the adaptive immune system. The author concluded that monocytes play a crucial role in mediating, distributing and transferring the immunomodulatory effect of MSC. It is undeniable that cellular interactions between MSC with cytotoxic T cells or monocytes are responsible for its immunosuppression function. Moreover, production of soluble factors by MSC plays an important role in immunosuppression procedure.^32^, ^33^ In our present study, these two mechanisms were integrated. Soluble factor TGF‐β1 could directly inhibit T cell proliferation; on the other hand, macrophage polarization was also observed. This is well in agreement with studies that demonstrated that phagocytosis of MSC induces an immunosuppressive phenotype in monocytes.^55^ In our present work, lentiviral vectors were used to delivery target TGF‐β1 gene in MSC. The risk of insertional mutagenesis associated with use of LV vectors should be concerned when it was implemented in clinical trials. In order to avoid such potential detriment, we want to adopt exosomes secreted by TGF‐β1 gene‐modified MSC instead of whole MSC infusion on our next research plan.

## 5 CONCLUSIONS

In conclusion, we have demonstrated for the first time that TGF‐β1 gene‐modified MSC showed enhanced alleviation of aGVHD severity in mice by skewing macrophages into a M2 like phenotype or increasing the proportion of Treg cells, which offers a novel option for the prevention and treatment of aGVHD.

## CONFLICT OF INTEREST

The authors declare that they have no conflicts of interest to disclose.

## AUTHORS' CONTRIBUTIONS

LM made substantial contributions to the design of the present study. RW performed the experiments, data collection and data analysis. RW and LM wrote the manuscript. CL, XD and LC provided help in conceiving and designing the study. SH made substantial contributions in data analysis. All authors read and approved the final manuscript.

## ETHICAL APPROVAL

All animal experiments were conducted according to guidelines published by The Ethics Committee of Xinhua Hospital Affiliated with the Shanghai Jiao Tong University School of Medicine. The protocols used in this study were approved by the Ethics Committee of Xinhua Hospital affiliated with Shanghai Jiao Tong University School of Medicine, Shanghai China.

## Data Availability

All data generated or analysed during this study are included in this published article.
